# VEGF-C is required for intestinal lymphatic vessel maintenance and lipid absorption

**DOI:** 10.15252/emmm.201505731

**Published:** 2015-10-12

**Authors:** Harri Nurmi, Pipsa Saharinen, Georgia Zarkada, Wei Zheng, Marius R Robciuc, Kari Alitalo

**Affiliations:** Wihuri Research Institute and Translational Cancer Biology Program, Biomedicum Helsinki, University of HelsinkiHelsinki, Finland

**Keywords:** cholesterol, lipid absorption, lymphatic vasculature, obesity, VEGF-C

## Abstract

Vascular endothelial growth factor C (VEGF-C) binding to its tyrosine kinase receptor VEGFR-3 drives lymphatic vessel growth during development and in pathological processes. Although the VEGF-C/VEGFR-3 pathway provides a target for treatment of cancer and lymphedema, the physiological functions of VEGF-C in adult vasculature are unknown. We show here that VEGF-C is necessary for perinatal lymphangiogenesis, but required for adult lymphatic vessel maintenance only in the intestine. Following *Vegfc* gene deletion in adult mice, the intestinal lymphatic vessels, including the lacteal vessels, underwent gradual atrophy, which was aggravated when also *Vegfd* was deleted. VEGF-C was expressed by a subset of smooth muscle cells adjacent to the lacteals in the villus and in the intestinal wall. The *Vegfc-*deleted mice showed defective lipid absorption and increased fecal excretion of dietary cholesterol and fatty acids. When fed a high-fat diet, the *Vegfc*-deficient mice were resistant to obesity and had improved glucose metabolism. Our findings indicate that the lymphangiogenic growth factors provide trophic and dynamic regulation of the intestinal lymphatic vasculature, which could be especially important in the dietary regulation of adiposity and cholesterol metabolism.

## Introduction

Lymphatic vessels regulate tissue fluid homeostasis, immune cell trafficking, and dietary fat absorption, and their malfunction leads to chronic edema and impaired immune responses (Cueni & Detmar, 2008; Alitalo, 2011; Koltowska *et al*, 2013). Lymphangiogenesis occurs during pathological processes such as inflammation and tumor metastasis and inhibitors of lymphangiogenic growth factors and their receptors are currently in clinical trials in human cancer patients (Alitalo, 2011). The development of the lymphatic vasculature is guided primarily by VEGF-C-mediated activation of VEGFR-3, which is the main VEGF receptor expressed by lymphatic endothelial cells (Makinen *et al*, 2001; Karkkainen *et al*, 2004). In the absence of VEGF-C, the development of lymphatic vessels is arrested during their initial spouting from embryonic veins (Karkkainen *et al*, 2004). VEGF-D, the second VEGFR-3 ligand, cannot compensate for the absence of VEGF-C during development, but it induces lymphangiogenesis when overexpressed and its deletion during development results in mild lymphatic vessel atrophy in the skin (Rissanen *et al*, 2003; Karkkainen *et al*, 2004; Alitalo, 2011; Paquet-Fifield *et al*, 2013; Astin *et al*, 2014).

During the postnatal period, the lymphatic vessels continue to expand, and in the intestine, the lacteal vessels grow into the intestinal villi to facilitate lipid absorption from the fat-rich milk (Kim *et al*, 2007). Lipid absorption allowed Gaspare Aselli to discover lymphatic vessels by their content of milky fluid in the 17^th^ century (Dixon, 2010). Recent studies have shown that the lacteal vessels are actively involved in lipid transport from the small intestinal epithelium to the lymphatic system and further to the blood circulation, although the detailed mechanisms have not been elucidated (Dixon, 2010). In several genetic mouse models of impaired lymphatic development, including *Vegfr3* and *Vegfc* hypomorphic mice, and *Chy* mice that have a missense point mutation in *Vegfr3*, lipid-rich chylous ascites develops after birth but resolves before weaning (Karkkainen *et al*, 2001, 2004; Haiko *et al*, 2008). However, the possible function of lymphangiogenic growth factors in the normally developed adult intestine is not known.

Here we have studied the role of VEGF-C in lymphatic vessel growth, maintenance, and function in neonatal and adult mice by using a mouse model that allows effective *Vegfc* gene deletion by the Cre-Lox system (Aspelund *et al*, 2014). We demonstrate that VEGF-C has a crucial role in the maintenance of the intestinal lymphatic vessels and dietary fat absorption.

## Results and Discussion

### *Vegfc* gene deletion arrests lymphatic vessel growth in the developing intestine

**Figure 1:**
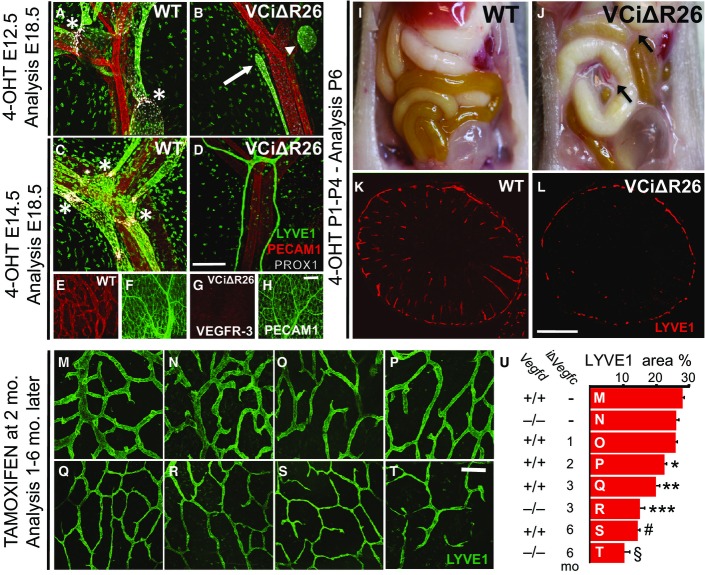
VEGF-C is required for lymphatic vessel growth in the developing intestine and for the intestinal lymphatic vessel maintenance in adult mice Mice received tamoxifen as indicated in the figure. Immunofluorescence analyses from the intestine were performed in (A–H) embryos, (I–L) pups, and (M–U) adults. A–D Mesenteric blood (PECAM1, red) and lymphatic vessels (LYVE1, green; PROX1, gray). Asterisks indicate lymphatic valves, arrow indicates a lymphatic vessel stub, and arrowhead indicates an isolated lymphatic vessel fragment

In *Vegfc* gene-deleted embryos, lymphatic vessel sprouting from the major embryonic veins at embryonic day (E) 10.5 is arrested and the embryos die between E15.5 and E17.5 (Karkkainen *et al*, 2004). To study how the loss of VEGF-C affects lymphatic vessel development during the last trimester of fetal development, we crossed the *Vegfc*^*flox/flox*^ mice (Aspelund *et al*, 2014) with mice expressing the universal deletor *R26Cre-ERT2* (Ventura *et al*, 2007). To delete *Vegfc* in the *R26Cre-ERT2;Vegfc*^*flox/flox*^ embryos, pregnant females were injected with 4-OH tamoxifen at E12.5 and E13.5. Analysis at E18.5 indicated that the *Vegfc*-deleted (VCiΔR26) embryos lack mesenteric lymphatic vessels either completely (data not shown) or exhibit lymphatic vessel fragments and blind-ended lymphatic stubs that extend toward the intestinal wall (Fig1A and B). Deletion of *Vegfc* at E14.5 blocked the maturation of the mesenteric collecting lymphatic vessels. The developing vessels lacked valves and were much thinner than in the wild-type (WT) *Vegfc*^*flox/flox*^ embryos (Fig1C and D). In addition, the lymphatic vessels in the intestinal wall failed to develop in the VCiΔR26 embryos, whereas the blood vessels were not affected (Fig1E–H).

E–H Blood vessels (PECAM1, green) and lymphatic vessels (VEGFR-3, red) in the small intestinal wall.

I, J Detection of chylous ascites (arrows) in the VCiΔR26 mice at P6.

K, L LYVE1 staining of intestinal lymphatic vessels at P6.

M–T LYVE1 staining of lymphatic vessels in the intestinal wall in adult mice. Genotypes and deletion lengths are indicated in (U).

U Quantification of LYVE1 areas in (M–T). Length of the *i*∆*Vegfc* gene deletion is indicated in months (mo), and *Vegfd* indicates the VEGF-D genotype. Data are represented as mean ± SEM. Significant differences were determined using one-way ANOVA and Bonferroni *post hoc* analysis compared to WT intestine represented in (M). *P = 0.003, **P = 0.001, ***P = 0.0008, ^♯^*P = *0.0002, ^§^*P = *0.0001. Data information: Scale bars: 200 μm in (A–H) and 400 μm in (K–T). *n *=* *25 (M); 5 (N); 8 (O); 7 (P); 6 (Q); 5 (R); 6 (S); 3 (T).

Lacteal lymphatic vessels develop after birth and are involved in the absorption of dietary lipids from milk (Kim *et al*, 2007). In order to determine whether VEGF-C is necessary for the formation of lacteal vessels, we deleted *Vegfc* by daily 4-OH tamoxifen injections to VCiΔR26 and WT pups from postnatal day 1 (P1) to P4. Upon necropsy at P6, chylous ascites was observed in the VCiΔR26 pups, but not in the WT littermates (Fig1I and J). Immunofluorescence staining of intestinal cross sections at P6 revealed that the lacteal vessels failed to develop in the VCiΔR26 pups (Fig1K and L and Appendix Fig S1A and B). Lymphatic vessels of the intestinal wall, which develop during the intrauterine period, failed to expand upon *Vegfc* deletion in postnatal period (Appendix Fig S1C–H). The lymphatic vessel density in the skin was also reduced in the VCiΔR26 pups when compared to their WT littermates (Appendix Fig S1I and J), whereas no changes were observed in the blood vessel density (Appendix Fig S1K and L). Taken together, these results indicate that VEGF-C is required for developmental and early postnatal lymphangiogenesis.

### VEGF-C is needed for lymphatic vessel maintenance only in the intestine

The VCiΔR26 mouse model allowed us to determine the role of VEGF-C in the maintenance of lymphatic vessels in adult mice. To induce *Vegfc* gene deletion, we administered tamoxifen to the mice starting at 8 weeks of age. Real-time PCR analysis of several tissues indicated that *Vegfc* mRNA levels in VCiΔR26 mice were reduced to 2–15% of the levels in WT mice 3 months after tamoxifen treatment (Appendix Table S1). Notably, whole-mount LYVE1 staining revealed that the lymphatic vessels of the intestinal wall undergo a gradual atrophy in the VCiΔR26 mice (Fig1M–U). Shorter lacteals were observed already 3 weeks after *Vegfc* gene deletion (Appendix Fig S2A), and after 3 months, the lacteals were strikingly thinner and shorter, containing a reduced number of lymphatic endothelial cells, suggesting that vessel atrophy was due to cell loss (Appendix Fig S2B). Thus, the lymphatic vessel atrophy started from lacteals and then progressed to the lymphatic network in the intestinal wall. In contrast, even when *Vegfc* was effectively deleted for 6 months, there were no changes in lymphatic vessel density in the lymph nodes, ears, or trachea (Appendix Fig S3A–E).

The development of the lymphatic vasculature relies principally on VEGF-C/VEGFR-3 signaling, while VEGF-D, the other known ligand for VEGFR-3, cannot compensate for the lack of VEGF-C signaling in *Vegfc*^−/−^ embryos (Karkkainen *et al*, 2004). Yet, VEGF-D was found to induce lymphangiogenesis when overexpressed in mouse tissues (Veikkola *et al*, 2001; Baluk *et al*, 2005). Constitutive deletion of *Vegfd* had no effect on lymphatic vasculature in the adult mouse intestine, in agreement with previous reports (Fig1U) (Baldwin *et al*, 2005). Surprisingly, however, conditional deletion of *Vegfc* in the *Vegfd*^−/−^ mice resulted in more severe atrophy of the intestinal lymphatic vessels than *Vegfc* deletion alone (Fig1M–U). These data indicate that VEGF-C is the primary trophic signal for the maintenance of the intestinal lymphatic vessels, but VEGF-D can partially compensate for the absence of VEGF-C. Because we did not observe changes in *Vegfd* mRNA expression levels upon *Vegfc* deletion (WT = 1.02 ± SEM 0.14 vs. VCiΔR26 = 0.98 ± SEM 0.16), it is likely that increased bioactive VEGF-D or increased VEGF-D binding to VEGFR-3 partially compensates for the loss of VEGF-C.

### VEGF-C is expressed by a subset of smooth muscle cells in the intestine

**Figure 2:**
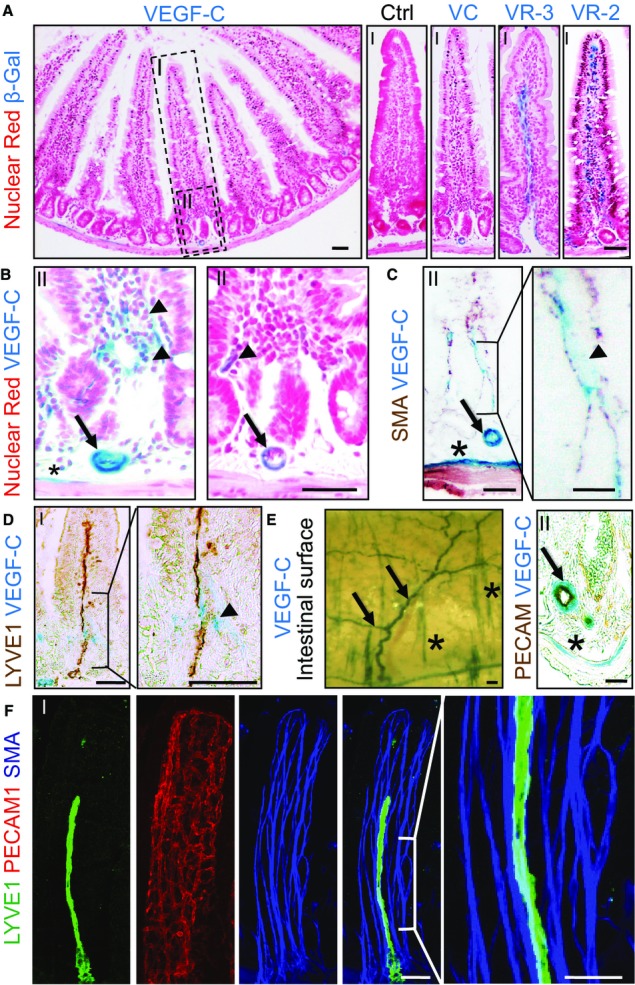
Smooth muscle cells are the main source of VEGF-C in adult intestine Overview of the small intestine cross section stained with nuclear red and highlighting the location of higher magnification images in (B–F); (I) for the entire villus and (II) for the villus base. β-Gal staining pattern of the villus in wild-type (Ctrl), *Vegfc*/LacZ (VC), *Vegfr3*/LacZ (VR-3), and *Vegfr2*/LacZ (VR-2) mice.

Next, we sought to identify the cell types that express VEGF-C in the adult intestine. Previous work from our laboratory showed that VEGF-C is expressed in arterial smooth muscle cells (SMCs) both in mice and humans (Partanen *et al*, 2000; Paavonen *et al*, 2002; Karkkainen *et al*, 2004). To define the cells expressing VEGF-C and its receptors, we performed β-Gal staining of intestines from *Vegfc*/LacZ (Karkkainen *et al*, 2004), *Vegfr3*/LacZ (Dumont *et al*, 1998), and *Vegfr2*/LacZ (Shalaby *et al*, 1995) mice. VEGF-C staining in the villi was weak in comparison with VEGFR-3 and VEGFR-2 stainings, in lymphatic and blood vessels, respectively (Fig2A and B). Higher resolution analysis combined with immunohistochemistry demonstrated VEGF-C expression in SMCs, in the inner circular muscle layer of the intestinal wall, in arterial smooth muscle, and in a subset of the SMC fibers in the villus (Fig2C). In the villi, the VEGF-C β-Gal signal was most prominent adjacent to the LYVE1-counterstained lymphatic vessels (Fig2D). The intestinal wall of the *Vegfc*/LacZ mice showed a prominent arterial β-Gal staining pattern, which was further analyzed by PECAM-1 counterstaining of cross sections (Fig2E), which confirmed VEGF-C expression in the arterial SMCs. Whole-mount confocal microscopy showed a close contact between the lacteal vessels and the SMC fibers in the basal part of the villus where also β-Gal staining of VEGF-C was detected (Fig2D and F). These results suggest that SMCs in the villi and in the intestinal wall provide an important source for VEGF-C, which is required to maintain the lymphatic vessel architecture in the intestine. It should be noted that SMC contractility in the villi has been suggested to be important for dietary lipid absorption and that VEGF-C can induce contraction of the SMCs around the collecting lymphatic vessels in normal and pathological conditions (Hosoyamada & Sakai, 2005, 2007; Breslin *et al*, 2007; Gogineni *et al*, 2013).

Higher magnification images representing β-Gal staining of the villus base in *Vegfc*/LacZ mice.

*Vegfc*/LacZ β-Gal staining reaction with smooth muscle actin (SMA) peroxidase staining.

*Vegfc*/LacZ β-Gal staining and LYVE1 peroxidase staining.

Surface image of *Vegfc*/LacZ β-Gal-stained intestine (left) and cross section counterstaining with PECAM1 (right).

Immunofluorescence staining of lacteal lymphatic vessel (LYVE1), blood capillaries (PECAM1), and smooth muscle cells (SMA). Data information: Arrows indicate the VEGF-C expression in arterial SMC, arrowheads indicate the VEGF-C expression in SMC fibers in the villus, and asterisks highlight the VEGF-C expression in circular smooth muscle cell layer of the intestinal wall. Scale bars: 50 μm, except (C) inset 25 μm.

To determine whether the effect of VEGF-C on intestinal lymphatic vessels is dependent on VEGFR-3, we analyzed the *Rosa26CreERT2;Vegr3*^*flox/flox*^ (R3iΔR26) mouse model (Haiko *et al*, 2008). As expected, deletion of *Vegfr3* for 3 months induced lacteal vessel regression, similar to VEGF-C deletion (Appendix Fig S4A and B). However, lymphatic vessel density in the intestinal wall was not altered, suggesting that VEGF-C can signal via VEGFR-2 to stabilize the lymphatic plexus. We have previously shown that the tyrosine kinase inhibitor cediranib inhibits lymphangiogenesis induced by adenoviral VEGF-C delivery into adult mouse skin (Heckman *et al*, 2008). However, administration of the tyrosine kinase inhibitor sunitinib at doses that block VEGFR-2 and VEGFR-3 (K_i_: VEGFR-3 17 nM/VEGFR-2 9 nM) (Faivre *et al*, 2007) had no effect on the lacteal vessels, although sunitinib significantly reduced blood vessel density in the intestinal villi (Appendix Fig S4C–E), in line with a previous report (Kamba *et al*, 2006). Thus, lacteal vessels appear to be more resistant than blood vessels in the intestinal villus toward VEGFR tyrosine kinase inhibition.

### *Vegfc* deletion reduces lipid absorption, inducing resistance to diet-induced obesity

To determine whether VEGF-C is important for lipid absorption by the intestinal lymphatic vessels, we performed an oral fat tolerance test in mice in which VEGF-C had been deleted 3 months earlier. The clearance of triglycerides from plasma was blocked by injection of Triton WR 1339, an inhibitor of lipoprotein lipase activity (Otway & Robinson, 1967). Analysis of triglyceride levels in serum after the administration of an oil bolus showed that the VCiΔR26 mice have impaired lipid absorption when compared to WT mice (Appendix Fig S5A). Although these results correlate with the lacteal regression, we cannot exclude the involvement of possible other effects of *Vegfc* deletion.

**Figure 3:**
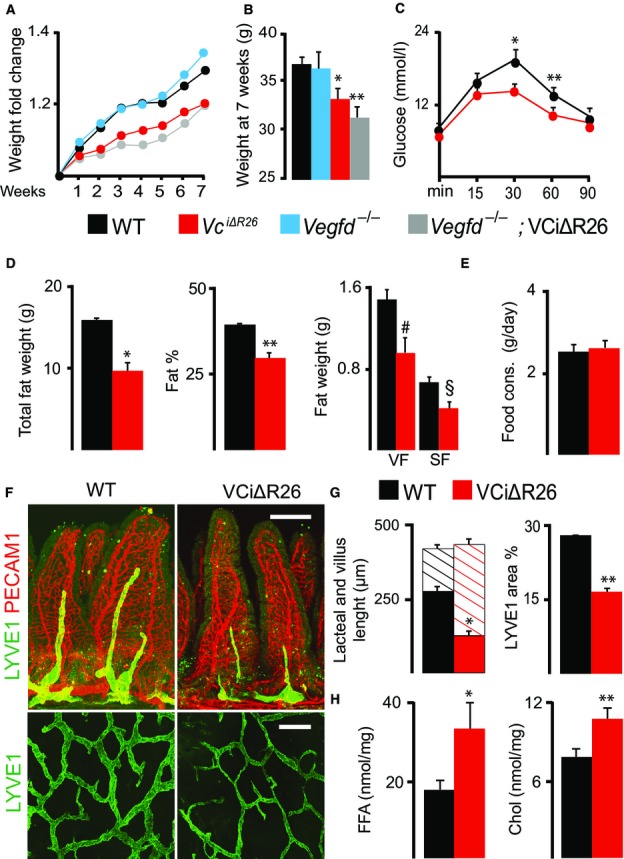
Intestinal lymphatic vessel regression leads to impaired lipid absorption and resistance to diet-induced obesity Two-month-old mice received tamoxifen and were fed on high-fat diet (HFD) for seven weeks before analysis. Body weight change during seven weeks of HFD, expressed as average fold change in comparison with the starting weight. *n *=* *16, WT; *n *=* *6, *Vegfd*^−/−^; *n *=* *16, VCiΔR26; *n *=* *5, *Vegfd*^−/−^; VCiΔR26.

We further studied whether the reduction in dietary lipid absorption observed in the VCiΔR26 mice has an impact on diet-induced obesity in mice fed high-fat diet (HFD). Initial experiments in the 129SV/C57Bl/6J mixed genetic background did not reveal major differences in body weight, but indicated that the VCiΔR26 mice have an improved glucose metabolism compared to WT mice (Appendix Fig S5B and C). Interestingly, in the mixed background, the *Vegfc*-deleted mice had reduced serum cholesterol levels, whereas fecal cholesterol and free fatty acid (FFA) levels were increased in the VCiΔR26 mice, indicating impaired dietary lipid absorption (Appendix Fig S5D). We further performed HFD feeding experiments in the pure C57Bl/6J background, an established model of diet-induced obesity. We deleted *Vegfc* in 8-week-old male mice and started HFD feeding 4 weeks later. The VCiΔR26 mice gained significantly less weight and had better glucose tolerance than their WT littermates, independently of concurrent *Vegfd* deletion (Fig3A–C and Appendix Fig S5E and F). At necropsy after HFD, very low amounts of chyle were detected in one out of 16 *Vegfc*-deleted mice and in two out of five *Vegfc-* plus *Vegfd*-deleted mice, indicating mild lymphatic leakage. Body composition analysis showed that the VCiΔR26 mice had a significant reduction in total fat weight and fat percentage, but no changes in lean weight in comparison with WT littermates (Fig3D and Appendix Fig S5G). The changes in fat accumulation could not be explained by reduced caloric intake, since food consumption was similar between the VCiΔR26 and WT mice (Fig3E). As expected on the basis of our results from the mixed background, *Vegfc* deletion induced intestinal lymphatic vessel atrophy and increased lipid excretion into the feces also in the C57Bl/6J background (Fig3F–H). No difference in body weight was observed between WT and *Vegfc*-deleted mice on regular diet in which the majority of calories are derived from carbohydrate. This further indicates that the reduced body weight of the *Vegfc*-deleted mice on HFD is a result of reduced dietary lipid absorption.

Body weight comparisons at seven weeks of HFD. Significant differences were determined using one-way ANOVA and Bonferroni *post hoc* analysis compared to WT. *P =* *0.004; **P =* *0.003. *n *=* *16, WT; *n *=* *6, *Vegfd*^−/−^; *n *=* *16, VCiΔR26; *n *=* *5, *Vegfd*^−/−^; VCiΔR26.

Glucose tolerance test (GTT) after six weeks of HFD. Significant differences were determined using unpaired two-tailed *t*-test. *P =* *0.014; **P =* *0.041. *n *=* *5, WT; *n *=* *6, VCiΔR26.

Total fat weight, fat percentage from body composition measurements after six weeks of HFD, and weights of visceral fat (VF) and subcutaneous fat (SF) at the time of necropsy. Significant differences were determined using unpaired two-tailed *t*-test. *P =* *0.006; **P =* *0.001; ^#^*P *=* *0.008; ^§^*P *=* *0.006. *n *=* *4 in each group.

Food consumption during the fifth week of HFD. *n *=* *9, WT; *n *=* *10, VCiΔR26.

Whole-mount immunofluorescence staining of blood (PECAM1, red) and lymphatic vessels (LYVE1, green) in intestinal villi and intestinal wall.

Quantification of the lacteal and villus length (solid and striped color bars, respectively) and the intestinal wall LYVE1^+^ area percentage from images represented in (F). Significant differences were determined using unpaired two-tailed *t*-test. *P =* *0.0002; **P =* *0.00007. *n *=* *5, WT; *n *=* *6, VCiΔR26.

Free fatty acid (FFA) and cholesterol measurements from the feces after six weeks of HFD. Significant differences were determined using unpaired two-tailed *t*-test. *P =* *0.001; **P =* *0.007. *n *=* *5, WT; *n *=* *6, VCiΔR26. Data information: Scale bars: 100 μm (villi) and 300 μm (intestinal wall). Data are represented as mean ± SEM.

Use of the newly established *Vegfc* gene targeted mouse model allowed us to determine the effect of chronic VEGF-C deficiency in adult mice, where lymphatic vasculature is normally in a quiescent state (Aspelund *et al*, 2014). The results of this study show that intestinal lymphatic vessels in adults require trophic signals from VEGF-C and that VEGF-D can only partially compensate for the loss of VEGF-C to maintain their structure and function. The unexpected finding that VEGF-C blockade affects only intestinal lymphatic vasculature and lipid absorption may provide new therapeutic opportunities. The specific VEGF-C/VEGFR-3 inhibitors that are currently in phase I clinical trials for cancer treatment could provide additional benefit for the treatment of obesity and cardiovascular disease by reducing the absorption of excess dietary lipids. Further studies should address lacteal vessel atrophy and dietary fat absorption in clinical trials employing VEGF-C/VEGFR-3 blocking therapeutics.

## Materials and Methods

### Study approval

National Animal Experiment Board in Finland approved all experiments involving the use of mice.

### Mice and tissues

The mouse lines *Vegfc*^*fl/fl*^ (Aspelund *et al*, 2014), *Vegfr3*^*fl/fl*^ (Haiko *et al*, 2008), *Rosa26-CreERT2* (Ventura *et al,* 2007*), Vegfd* (Baldwin *et al*, 2005), *Vegfc*/LacZ (Karkkainen *et al*, 2004), *Vegfr3*/LacZ (Dumont *et al*, 1998), and *Vegfr2*/LacZ (Shalaby *et al*, 1995) have been described previously. We used VCiΔR26 mice in the mixed C57Bl/6J and 129SV background or after backcrossing to the C57Bl/6J strain for at least 7 generations. For induction of Cre-mediated recombination in embryos, the mother was injected at the indicated days with two consecutive intragastric doses of 4-OH tamoxifen (4-OHT) (Sigma) (25 mg/ml dissolved in 100 μl ethanol/olive oil). In the neonatal VCiΔR26 or WT mice, the Cre-mediated recombination was induced between P1 and P4 by daily intragastric administration of 2 μl 4-OHT (25 mg/ml dissolved in ethanol). Recombination in adult mice (7–8 weeks old) was done by intragastric tamoxifen (Sigma, dissolved in corn oil at 2 mg/ml, 100 μl) administration during five consecutive days. Detailed animal experiment information can be found in the Appendix Supplementary Materials and Methods.

### Statistics

Quantitative data were compared between groups by two-tailed unpaired *t*-test or one-way ANOVA followed by Bonferroni *post hoc* test for multiple comparisons. Values are expressed as mean ± SEM. *P*-value < 0.05 was considered significant.

## Supporting Information

**Appendix**

**Review Process File**
