# Supplementary material for: VEGF-C is required for intestinal lymphatic vessel maintenance and lipid absorption
Source: EMBO Mol Med. 2015 Oct 12;7(11):1418–25. doi: 10.15252/emmm.201505731 (PMC4644375; doi:10.15252/emmm.201505731)
Supplement: Supplementary file 1 [file emmm0007-1418-sd1.pdf]

VEGF-C is required for intestinal lymphatic vessel structure and function

Harri Nurmi et al., Appendix

Table of Contents:

Appendix Figure and table legends

Appendix Supplementary Materials and Methods

Appendix Figures 1-5 and Appendix table S1

Appendix Figure Legends

Appendix Figure S1. VEGF-C is required for postnatal lymphangiogenesis. VCiΔR26 and WT littermates were injected with 4-OH tamoxifen between P1 to P4 and analyses were performed at P6. Immunoflurescence staining of Prox1 (Green) and VEGFR3 (Red) in the villi

(**A**) Arrows indicate lymphatic vessels in the intestinal wall. Whole mount immunofluorescence staining of blood vessels (PECAM1, red) and lymphatic vessels (LYVE1, green) in the intestinal wall **(C-D and F-G)** and skin (**I-L**)

Quantification of Lyve1 **(E)** and Pecam1 **(H)** area from the intestinal wall stainings. **(E)** Significant differences were determined using two-tailed unpaired t-test. **P* = 0.0002, N=5/group.

Scale bars 50 μm (A-B), 200 μm (C-D and F-G) and 300 μm (I-L). Data are represented as mean ± SEM.

Appendix Figure S2. Deletion of *Vegfc* causes lacteal shrinking. (A) Whole mount staining of lymphatic vessels in intestinal villi after three weeks of *Vegfc* deletion and quantifications of the lacteal and villus lengths (solid and striped color bars respectively). Significant differences were determined using two-tailed unpaired t-test. * *P* =0.03, N=6 (WT) N=8 (VCiΔR26) **(B)** Whole mount staining of lymphatic vessels in intestinal villi and LEC/lacteal quantification after three months of *Vegfc* deletion. Significant differences were determined using two-tailed unpaired t-test. * *P* =0.002, N=6 (WT), N=6 (VCiΔR26).

Scale bars 50 µm (A-B) Data are represented as mean ± SEM.,

Appendix Figure S3. Analysis of lymphatic and blood vasculature after long-term *Vegfc* deletion in adults. (A) Inguinal lymph nodes stained with LYVE1. **(B)** Staining of intestinal blood vessels after 3 months of *Vegfc* deletion. **(C)** Staining of lymphatic vessels in the skin and trachea 6 months after *Vegfc* deletion. **(D)** Quantification of blood and lymphatic vessel areas from images represented in panels B and C. **(E)** Relative VEGF-C mRNA levels in the intestine, skin and trachea. Significant differences were determined using two-tailed unpaired t-test. * *P* =0.006 (Intestine); * *P* =0.0009 (Skin); * *P* =0.0003 (Trachea). N=4 (WT), N=5(VCiΔR26).

Scale bars 300 µm. Data are represented as mean ± SEM.

Appendix Figure S4. VEGFR-3 removal causes lacteal vessel shortening and RTK-inhibition reduces blood vessel density (A) Immunofluorescence staining of blood vessels (PECAM1, red) and lymphatic vessels (LYVE1, green) in the intestinal villi and intestinal wall. **(B)** Quantifications of the lacteal and villus lengths (solid and striped color bars respectively) and Lyve1 area % in the intestinal wall. Significant differences were determined using two-tailed unpaired t-test. * P=0.002, N=4/group **(C)** Representative whole mount staining of the villus images from Control and Receptor tyrosine kinase-inhibitor Sunitinib (60mg/kg – 14 Days) treated mice. **(D)** Quantifications of the lacteal and villus lengths (solid and striped color bars respectively). N=5 (Sunitinib) N=4 (Control) **(E)** PECAM1 area quantification from villus. Significant differences were determined using two-tailed unpaired t-test. * P =0.02, N=5 (Sunitinib) N=4 (Control).

Scale bars 100 upper A and C 100 μm, lower A 200 μm. Data are represented as mean ± SEM.

Appendix Figure S5. Impaired lipid absorption in the *Vegfc* deleted mice.

(A) Triglyceride concentration in serum after administration of an oil bolus to VCiΔR26 and WT mice in C57bl/6J genetic background maintained on chow diet. Significant differences were determined using two-tailed unpaired t-test. * P =0.023. N=5 (WT) N=6 (VCiΔR26)

(B) Body weights of VCiΔR26 and control mice in mixed genetic background during 12 weeks of HFD.

(C) Intra-peritoneal (IP) glucose (1g/kg glucose, IP-GTT) and insulin tolerance tests (0.75 U/kg insulin, IP-ITT), after 12 weeks of HFD in mixed background. Significant differences were determined using two-tailed unpaired t-test. * *P* <0.05. N=6 (WT) N=7 (VCiΔR26).

(D) Fasting cholesterol concentration from blood, and cholesterol (Chol) and free fatty acid (FFA) concentration in the stools**.** Significant differences were determined using two-tailed unpaired t-test. * *P* =0.003 (Blood) * *P* =0.007 (Stool Chol) * *P* =0.0008 (Stool FFA). N=6 (WT), N=7 (VCiΔR26)

(E) Fold increase of body weight during seven weeks of HFD in C57bl/6J background mice. Significant differences were determined using two-tailed unpaired t-test. * *P* =0.002, N=8 (WT) N=10 (VCiΔR26)

(F) Area under curve (A.U.C) from the IP glucose tolerance test. Significant differences were determined using two-tailed unpaired t-test. * *P* =0.03, N=8 (WT) N=10 (VCiΔR26)

(G) Lean weight based on body composition measurements. (A and E-G) Pure C57bl/6J background mice and (B-D) Mixed background mice.

Appendix Table S1. *Vegfc* mRNA levels in the indicated tissues three months after TAM induced *Vegfc* gene deletion.

Appendix Supplementary Materials and Methods

Mice and tissues. For embryonic and postnatal analyses, the mice were sacrificed by decapitation and the tissues were immersed in 4% paraformaldehyde, washed with PBS and prepared for whole mount staining or embedding into OCT. Tissues from adult animals were collected after lethal anesthesia followed by cervical dislocation.

Experimental setups: Treatment with the receptor tyrosine kinase (RTK)-inhibitor Sunitinib was done by daily intra-gastric administration in concentration of 60mg/kg for 14 days. Sunitinib was diluted to 1,8% NaCl, 0,1 % Tween20, 0,5 carboxy-methyl cellulose solution. Control treated mice were administrated the same solution without Sunitinib.

For the HFD experiments, the mice were treated with tamoxifen at the age of 7-8 weeks. The diet (60 %, D12492, Research Diets Inc.) was started approximately 4 weeks later and continued for additional 8 weeks. The glucose tolerance test (1g/kg; GTT) and the insulin tolerance test (0,75U/kg; ITT) were performed after 6 hours of fasting. The feces were collected for lipid analysis. Mouse body composition was analyzed by dual-energy x-ray absorptiometry (DEXA) densitometry (Lunar PIXImus 2, GE Heathcare) after 8 weeks of HFD. For lipid absorption test, mice were pre-treated with WR1339 (Tyloxapol;Sigma) 500 mg/kg i.p for lipase inhibition one hour prior oil Gavage.

LEC quantification was done by counting Lyve1+; DAPI positive endothelial cells in the villus.

Antibodies. The following primary antibodies were used for tissue immunostaining: goat anti-human PROX1 (diluted 1:500; AF2727, R&D Systems), polyclonal goat anti-mouse VEGFR-3 (diluted 1:100; AF743, R&D Systems), rat anti–PECAM-1 (diluted 1:500; clone MEC 13.3, 553370, BD Biosciences — Pharmingen), polyclonal rabbit anti–LYVE-1 (diluted 1:1,000; ref. (5)) mouse anti-SMA (Cy3-conjugated, clone 1A4, C6189, Sigma). Primary antibodies were detected with the appropriate Alexa Fluor 488, Alexa Fluor 594, Alexa Fluor 633 or Alexa Fluor 647 secondary antibody conjugates (diluted 1:300; Molecular Probes/Invitrogen).

For β-galactosidase staining reaction intestinal samples were perfused with fixative containing 1% Formalin; 0,2 % Glutaraldehyde and 0,02% NP-40 in PBS. 2 h fixation was done at +4 °C with gentle mixing followed by X-Gal (1mg/ml) reaction in room temperature over night.

Microscopy. Immunofluorescence images were taken with a Zeiss LSM780 confocal microscope (10x NA:0.45; 20X NA:0.80) or Zeiss Axioplan 2 microscope (5x NA:0.15;10x NA:0.3) (Carl Zeiss AG). Bright-field sections were viewed with a Leica DM LB microscope (Leica Microsystems) and images were captured with an Olympus DP50 color camera (Olympus Soft Imaging Solutions GMBH).

Lipid analysis. The collected feces were left to dry for 24 hours, weighted and dissolved in chloroform for homogenization. The lipids were then separated by using Chloroform:Methanol (2:1) extraction, followed by solvent evaporation in a heated vacuum. The lipids were dissolved into 5% Triton-X in PBS for further analysis. Cholesterol and triacylglycerol concentrations were determined with enzymatic methods (Roche Diagnostics Hitachi). Free fatty acid levels were measured with NEFA R2 kit downscaled to microplate format (Wako).

Real-time quantitative PCR. Total RNA was extracted and isolated from indicated tissues using the NucleoSpin®RNA II Kit (Macherey-Nagel) according to the manufacturer's protocol. To eliminate contaminating DNA, RNase-free DNase I (lyophilized) was used during RNA isolation. Samples were quality-controlled using a Nanodrop ND-1000 or BioSpec-nano spectrophotometer. Reverse transcription into cDNA was performed with 1 µg of total RNA using iScript™ cDNA Synthesis Kit (Bio-Rad). Real-time quantitative PCR (RTqPCR) was performed using TaqMan Gene Expression Assays (Applied Biosystems) and the iQ™ Supermix kit (Bio-Rad). RT-qPCR was carried out using a BIO-RAD C1000 Thermal cycler according to a standardized protocol. The TaqMan Gene Expression Assays used for mouse mRNA were *Vegfc* (Mm00437310_m1) and Gapdh (4352932E). The data were normalized to the endogenous control Gapdh to compensate for experimental variations. Fold changes were calculated using the comparative CT method.

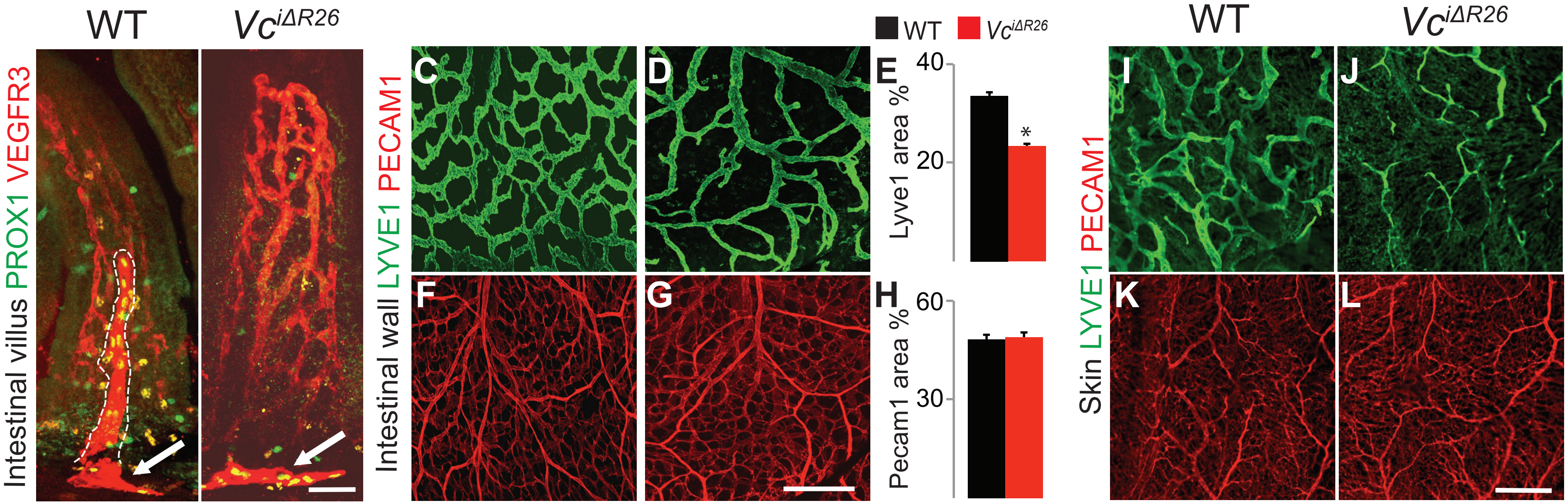

Nurmi et al, Figure S1

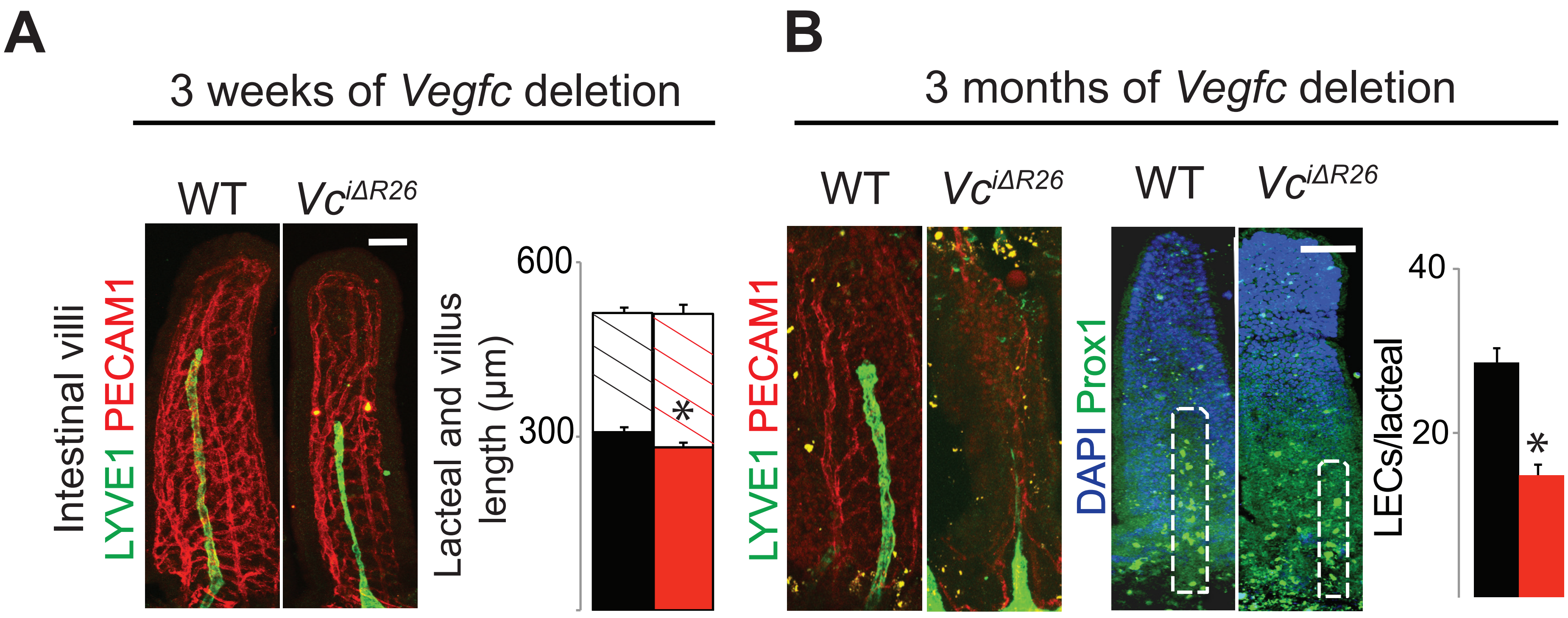

Nurmi et al., Figure S2

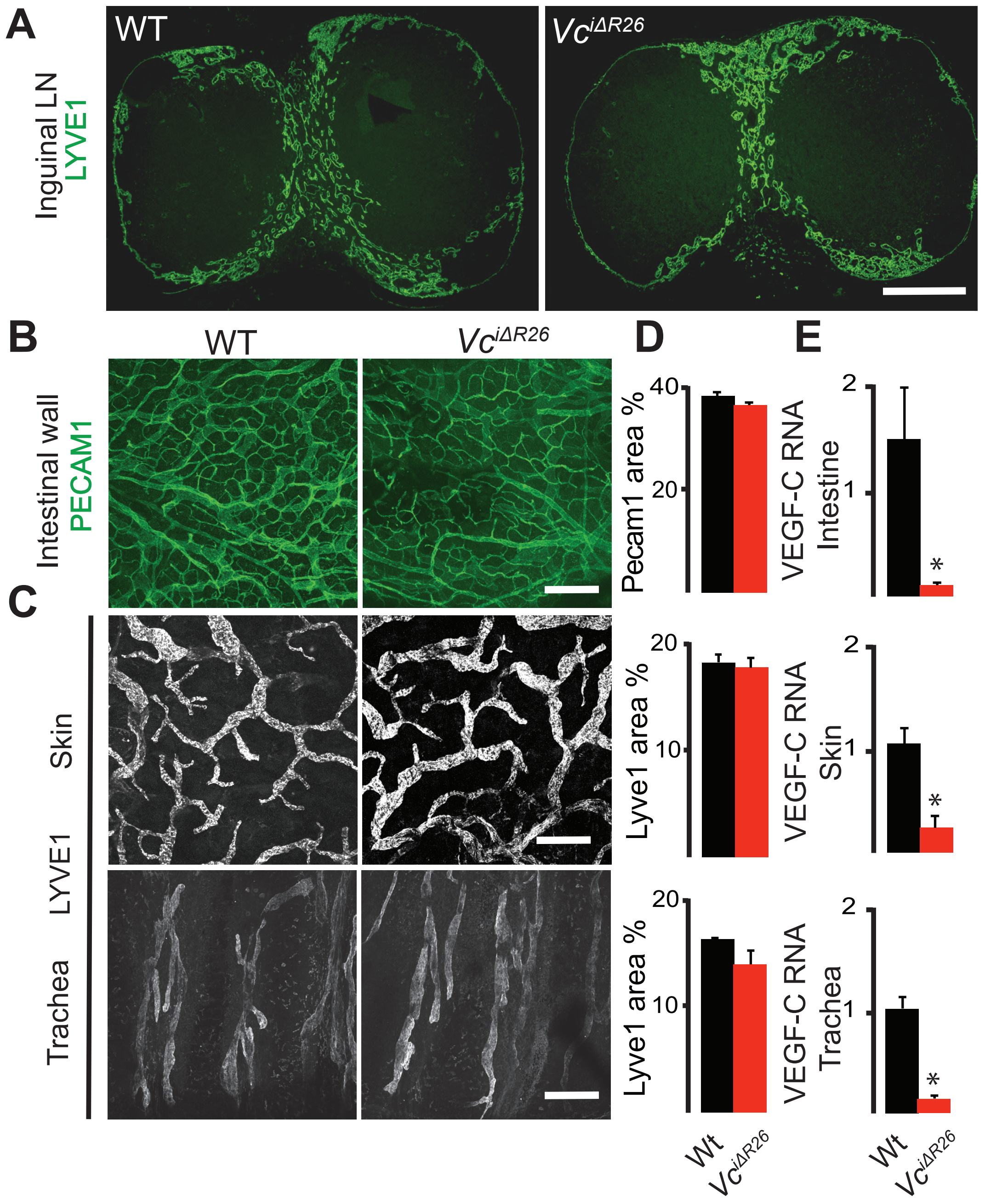

Nurmi et al., Figure S3

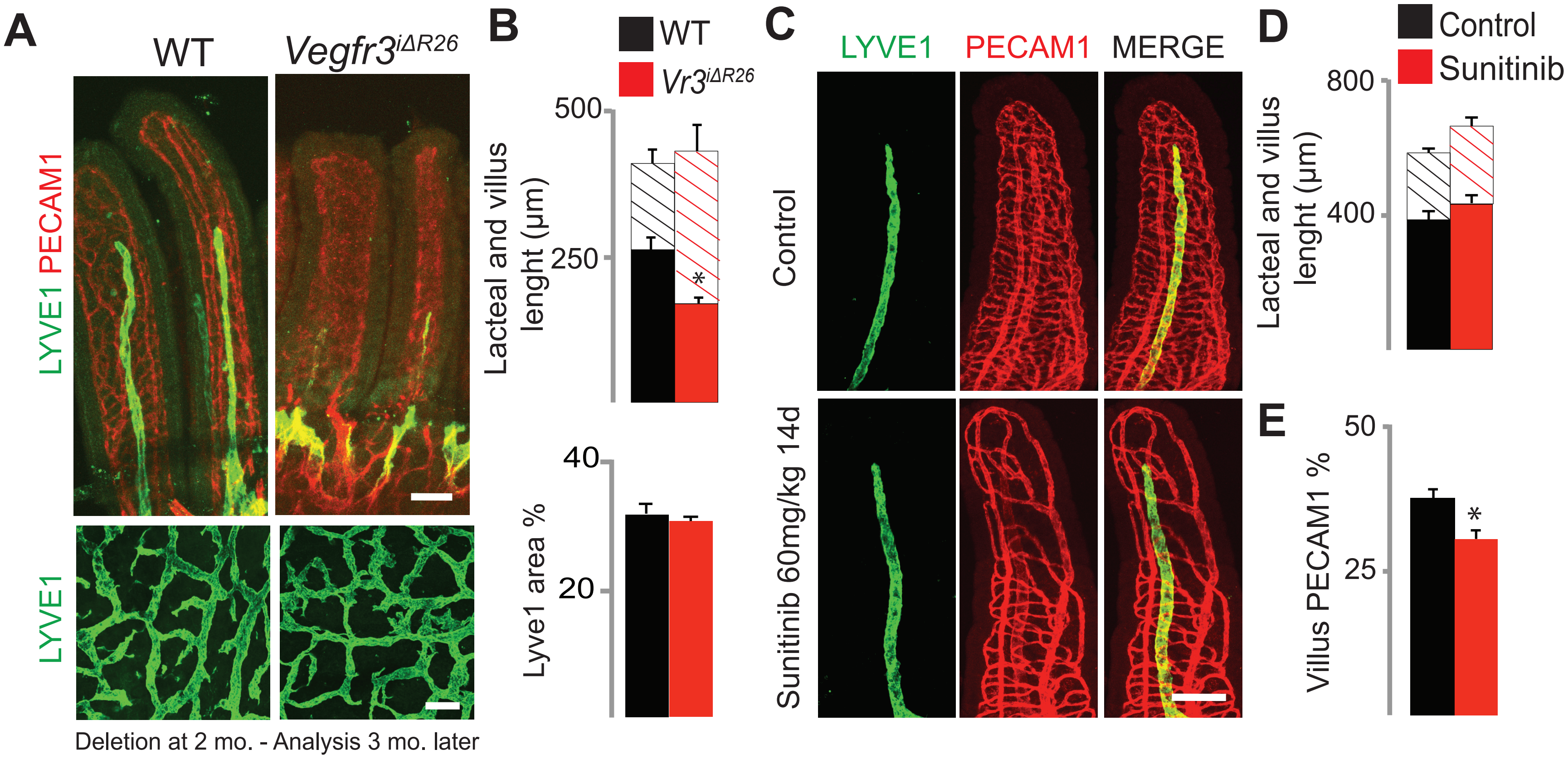

Nurmi et al., Figure S4

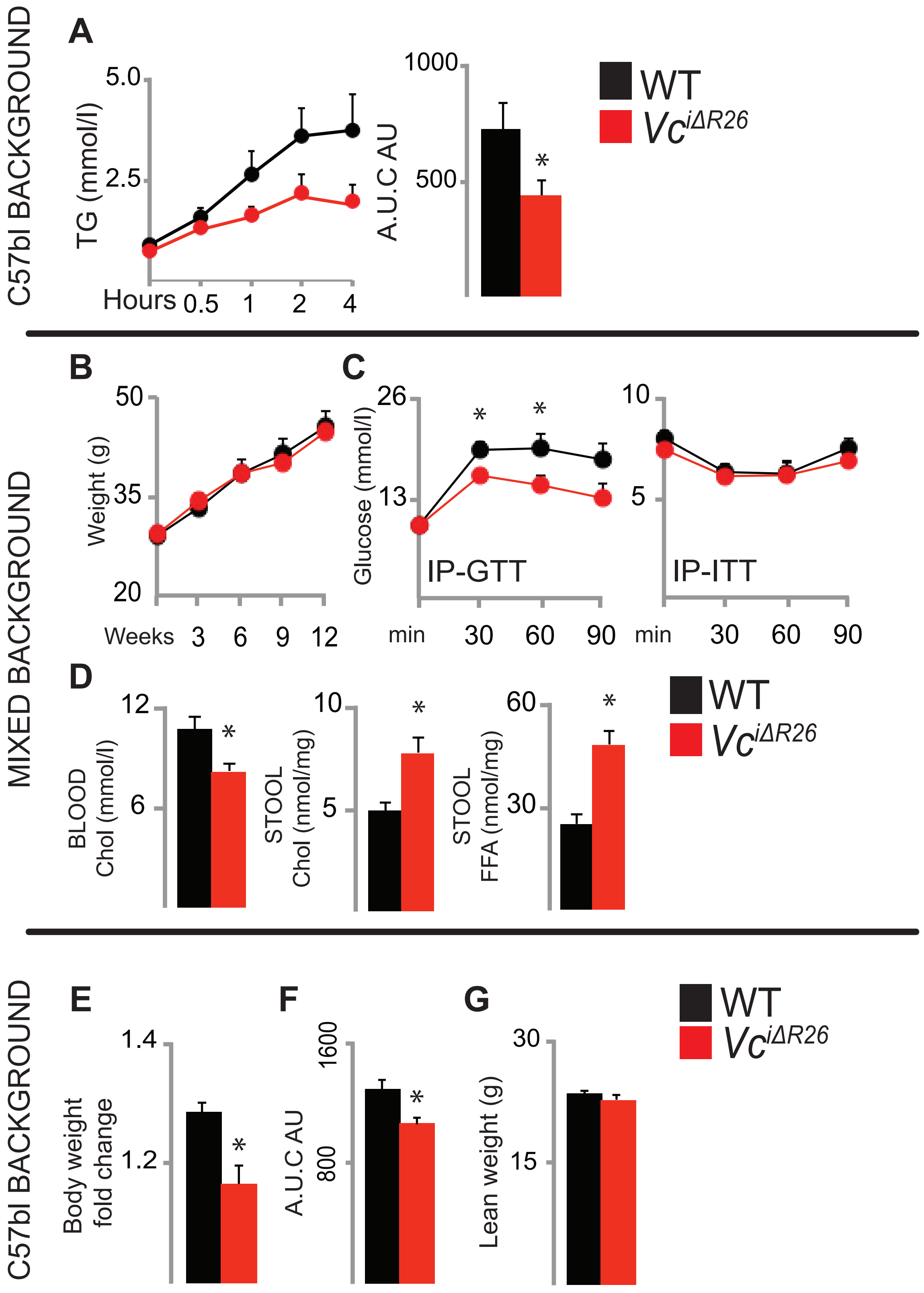

Nurmi et al., Figure S5

Appendix table S1

Analysis of *Vegfc* mRNA levels by Real Time PCR in indicated tissues three months after tamoxifen administration.

**Tissue**	Wild Type	$Vc^{i\Delta R26}$
Diaphragm	1.00 ± 0.071	0.09 ± 0.038 *
Heart	1.02 ± 0.125	0.02 ± 0.004 *
Intestine	1.38 ± 0.422	0.12 ± 0.053 *
Liver	1.10 ± 0.196	N.D
Lung	1.00 ± 0.052	0.04 ± 0.028 *
Ovary	1.03 ± 0.163	0.02 ± 0.005 *
Skin (Ear)	1.05 ± 0.143	0.15 ± 0.052 *
Testis	1.03 ± 0.122	0.12 ± 0.085 *
Trachea	1.02 ± 0.115	0.15 ± 0.026 *

Values were normalized to *Gapdh* and expressed relative to WT
*P < 0.05; N.D = Not Detectable N:3-8/tissue
