# Supplementary material for: VEGF-C is required for intestinal lymphatic vessel maintenance and lipid absorption
Source: EMBO Mol Med. 2015 Oct 12;7(11):1418–25. doi: 10.15252/emmm.201505731 (PMC4644375; doi:10.15252/emmm.201505731)
Supplement: Supplementary file 2 [file emmm0007-1418-sd2.pdf]

EMBO
Molecular Medicine

VEGF-C is required for intestinal lymphatic vessel maintenance and lipid absorption

Harri Nurmi, Pipsa Saharinen, Georgia Zarkada, Wei Zheng, Marius R. Robciuc and Kari Alitalo

Corresponding author: Kari Alitalo, Biomedicum Helsinki Faculty of Medicine

Review timeline:

Submission date:	07 August 2015
Editorial Decision	09 September 2015
Follow up on Editorial Decision:	11 September 2015
Revision received:	22 September 2015
Accepted:	25 September 2015

Transaction Report:

(Note: With the exception of the correction of typographical or spelling errors that could be a source of ambiguity, letters and reports are not edited. The original formatting of letters and referee reports may not be reflected in this compilation.)

Editor: Roberto Buccione

1st Editorial Decision 09 September 2015

Thank you for the submission of your manuscript to EMBO Molecular Medicine. We are sorry that it has taken longer than usual to get back to you on your manuscript.

We are still missing one evaluation. To avoid a further delay, I have decided to proceed based on these available consistent evaluations. I should inform you that if in the meanwhile we should receive the third evaluation, but only if it raises significant caveats, these would need to be taken into consideration. We would not, however, ask you to comply with any further-reaching requests.

As you will see, both Reviewers are quite appreciative of the manuscript and suggest a number of improvements that should work towards increasing impact, clarity and conclusiveness. I will not go into detail as the comments appear clear and should not entail major difficulties. I would like however to emphasise Reviewer 1's question on whether the long-term use of anti-VEGFR3-targeted therapies is likely to affect dietary uptake and the nature of intestinal lymphatic vessels and the likely consequences of prolonged targeting of VEGF-C (or VEGFR3) in limiting intestinal lipid absorption. As this Reviewer mentions, it would indeed be valuable for our readership if you could place your findings in perspective.

In conclusion, while publication of the paper cannot be considered at this stage, we would be pleased to consider a suitably revised submission, with the understanding that the Reviewers' concerns must be fully addressed and that acceptance of the manuscript might entail a second round of review.

Please note that it is EMBO Molecular Medicine policy to allow a single round of revision only and that, therefore, acceptance or rejection of the manuscript will depend on the completeness of your responses included in the next, final version of the manuscript.

As you know, EMBO Molecular Medicine has a "scooping protection" policy, whereby similar findings that are published by others during review or revision are not a criterion for rejection. However, I do ask you to get in touch with us after three months if you have not completed your revision, to update us on the status. Please also contact us as soon as possible if similar work is published elsewhere.

Please note that EMBO Molecular Medicine now requires a complete author checklist (http://embomolmed.embopress.org/authorguide#editorial3) to be submitted with all revised manuscripts. Provision of the author checklist is mandatory at revision stage; The checklist is designed to enhance and standardize reporting of key information in research papers and to support reanalysis and repetition of experiments by the community. The list covers key information for figure panels and captions and focuses on statistics, the reporting of reagents, animal models and human subject-derived data, as well as guidance to optimise data accessibility.

I suggest that you carefully adhere to our guidelines for publication in your next version, including presentation of statistical analyses and our new requirements for supplemental data (see also below) to speed up the pre-acceptance process.

I look forward to seeing a revised form of your manuscript as soon as possible.

***** Reviewer's comments *****

Referee #1 (Remarks):

General

The manuscript by Nurmi H. et al. describes the phenotype of Vegfc gene deletion in adult mice. General deletion of Vegfc using the R26Cre-ERT2 driver mouse line surprisingly only affected intestinal lymphatic vessels, resulting in vessel atrophy, resulting in defective lipid absorption and increased excretion of dietary cholesterol and fatty acids. Furthermore, this perturbed intestinal lipid transport protected Vegfc-deficient mice from obesity when placed on a high-fat diet. The work presented here is of great interest for the EMBO Mol Med readership, and furthers our understanding of the factors that regulate lymphatic vessel maintenance and function in postnatal health.

Comments:

1) The statement on page 5 that Vegfc gene-deleted embryos "die between embryonic days (E) 15.5-17.5, soon after the first lymphatic endothelial cells emerge from embryonic veins" should be clarified in light of the fact that the first lymphatic precursors emerge from the cardinal vein at E10.5.

2) What is the penetrance and severity of VCiΔR26 phenotypes for the various 4-OH tamoxifen administration regimens?

3) Are any other lymphatic vascular beds affected during embryonic development as a result of deletion of Vegfc? Given the recent papers on lymphatics in the brain and heart, does loss of Vegfc

affect lymphatics in these organs, and is development of these organs also affected?

4) For the LYVE1 staining of the lacteal (lymphatic) vessels in Fig. 1K,L, a second lymphatic marker would be beneficial to demonstrate that lymphatic vessels do not simply have reduced LYVE1 immunoreactivity as a result of loss of Vegfc.

5) On page 6, it is unclear what is meant by "six-day postnatal Vegfc deletion" where "no changes were seen in the lymphatic vessels that develop during the intrauterine period" (referring to S. Fig. 1E-H). In the micrographs provided, panel F appears to have a reduced lymphatic area, with thinner vessels that have less visible branch points (compared to S. Fig. 1E). Can this be clarified.

6) On page 7, it is stated that there were "thinner and shorter lacteal vessels with a reduced number of lymphatic endothelial cells", and "the slow lacteal regression after genetic deletion of Vegfc occurs primarily by the loss of LECs via cell death". How was this assessed?

7) The statement that "VEGF-D can partially compensate for the absence of VEGF-C" is intriguing. Is there an upregulation of VEGF-D in VCiΔR26 mice?

8) Is the long-term use of anti-VEGFR3-targeted therapies likely to affect dietary uptake and the nature of intestinal lymphatic vessels? What are the likely consequences of prolonged targeting of VEGF-C (or VEGFR3) to limit intestinal lipid absorption? It would be interesting for the EMBO Mol Med readership to place these interesting findings in perspective.

Minor comments

1. Immunostaining for Prox1 (white) in Fig. 1A-D is rather difficult to see in the micrographs provided, and should be highlighted with visual cues (i.e. asterisks) or changed to another color.

2. Additional labels should be provided in Fig. 1 to clarify which panels are WT and VCiΔR26. Are panels C,E,F WT and D,G,H VCiΔR26?

3. The quality of the brightfield micrographs in Fig. 2 can be improved. The SMA-positive SMCs are difficult to detect, with respect to the architecture of the vessel wall and surrounding tissues. Immunofluorescence labeling of the lymphatic endothelium, along with staining for SMA and ß-Gal may improve visualization of VEGF-C localization.

4. The quality of the PECAM1 staining in Fig. 2E is poor, and it is difficult to discern the organization of blood vessels, especially compared to the micrographs in Fig. 3F.

5. The graphs in Fig. 3 lack a line delineating the X-axis, and it would be easier for the reader to follow if the black and red bars were labeled in each graph.

6. On page 14, "CONFLICT OF INTREST" should be "CONFLICT OF INTEREST".

7. For S. Fig. 2B, Title: "Inquinal" should be "Inguinal"

8. Scale bars are missing from Fig. 2B (plus inset), 2C (inset), 2D and 2E; S. Fig. 2E (skin).

9. Some of the journal abbreviations in the references are not correctly formatted (#10, 22, 23).

Referee #3 (Comments on Novelty/Model System):

In this manuscript the Alitalo group expand on their previous excellent research on the mechanisms regulating lymphangiogenesis. Having previously demonstrated that VEGFC signaling is required for the migration and survival of lymphatic endothelial cells forming the lymph sacs, they now make use of an inducible VEGF-C knockout mouse model to investigate the role of VEGF-C in lymphangiogenesis during later stages of development. The main finding is that VEGF-C is required for developmental and early postnatal lymphangiogenesis in the intestine and plays a role in the long term maintenance of the lymphatic vessels in the intestine but not other organs (skin, trachea, lymph nodes) of adult animals. Additional interesting observations were that the phenotype of vegfc deletion was aggravated when Vegfd was also deleted and that Vegfc deleted mice showed defective

lipid absorption rendering them more resistant to obesity and glucose intolerance when subjected to a high fat diet.

Referee #3 (Remarks):

The manuscript provides novel and interesting insights into the dynamic function of VEGFC in the regulation of the intestinal lymphatic vasculature. The maintenance function described for VEGFC in the intestinal lymphatic vasculature and its impact on lipid absorption represent exciting new aspects and opportunities for future studies in this field. The manuscript is concise and generally well written and the results are adequately presented in the figures. Having said this, on occasion the comparison between panels showing different experimental settings (embryonic vs postnatal vs long term deletion) different genetic backgrounds (pure vs mixed) in various organs (intestine, skin, trachea) and different types of analysis is somewhat confusing and hard to follow, especially when several variables change even in one figure or the results are split between the main figure and supplement. Nevertheless, the quality of the research is of a very high standard and the figures were carefully compiled.

Major Issues:

I have no major concerns.

Minor Issues:

Fig. 1: panels M-T are difficult to interpret without reference to panel U. Perhaps the authors could label these panels more clearly?

Fig. S1A/B: could the authors include a brief comment in the text on the observation that the lymphatic vessel density was reduced in the skin?

Fig. 2A: The difference in expression patterns of VEGFC vs VEGFR2 can be recognized but its not terribly clear. While I appreciate that technical difficulties are partly responsible, it might also help to show the tip of the villus to more easily relate the staining pattern to the precise position within the villus.

In Fig. 2B/C Given the technical difficulties in staining VEGFC, have the authors considered other markers of smooth muscle cells except SMA? Also labelling of the different structures stained (arterial SM, circ. muscle layer etc.) would be helpful for more rapid interpretation. The villus outline is hard to recognize without nuclear red counterstain (for obvious reasons), so perhaps this could be outlined instead?

Fig. S2A: Again, the outline (tip to base) of the villus is not visible, making interpretation of the LYVE1 signal difficult. The Prox1 staining pattern shows a lot of background, and overall little difference between the two panels -the stippled box appears to be placed rather arbitrarily.

Fig. S2B The type of staining should be added to the figure.

Fig. S3C: The lack of lacteal regression in response to sunitinib is a very interesting and surprising finding, especially in comparison with the dramatic effects shown for VEGFR3 deletion. Given that

Sunitinib is rather nonspecific, the use of more specific RTKIs might help to pinpoint the molecular signals involved. Has/could this been/be explored?

Fig. S4D: please label which samples are serum and stool, respectively.

Fig. S4E: this is based on a pure background of mice, while the other data is from mixed background - please indicate this in the figure as well as in the legend.

How do the authors explain the different response in terms of weight gain upon high fat diet in mixed (Fig. S4B) vs pure background (Fig. 3A) animals given similar GTT results upon VEGFC deletion? Have the authors looked for any differences in lymphatic vessel atrophy in the pure vs mixed background mice?

The observation that VEGFc deleted mice appear to be resistant to high fat diet is of potential clinical value. In particular because in these mice only the intestinal lymphatic vessels are affected without compromising the remaining lymphatic system as underlined by the lack of significant accumulation of chyle.

Follow up on Editorial Decision 11 September 2015

I have now received the missing evaluation from Reviewer 2. As you will see this is quite positive too and lists a few items that do not appear difficult to deal with and which I would suggest you do in your revision.

Provided you deal with the reviewers' comments carefully and completely, I am prepared to make an editorial decision. Do allow me to reiterate that it is essential that you comply with all the editorial requirements listed immediately below to ensure a speedy process upon receipt of the revision.

I look forward to reading a revised version of your manuscript as soon as possible.

***** Reviewer's comments *****

Referee #2 (Remarks):

Nurmi et al expand analysis of a recently described conditional Vegf-C allele that allows inducible deletion of this critical lymphangiogenic growth factor. Generation and characterization of a conditional Vegf-C allele is an important addition to the toolkit available to study the function of this important growth factor and the findings should be of wide interest to the angiogenesis community.
The generation of the allele, and description of lymphatic aplasia in the embryonic skin was published in JCI 2014, here the authors focus on adult deletion of Vegf-C using a tamoxifen-inducible ubiquitous Cre driver line. They show that adult deletion of Vegf-C preferentially targets lacteals, which are terminal lymphatic vessels within the intestinal villi that are responsible for uptake of dietary fat. Interestingly, they show that absence of lacteals prevents dietary fat uptake and renders mice resistant to diet-induced obesity, without apparent deleterious effects on mouse health. Very little is known about the mechanisms controlling chylomicron uptake into lacteals, this paper shows for the first time that lacteals are indispensable for dietary fat uptake and that preventing lacteal growth may be a novel avenue to combat obesity.
I find the paper of great interest for Embo Molecular Medicine and have only a few minor comments that the authors should address before publication.

Fig.1: is inducible deletion of Vegf-C equivalent to global loss of Vegf-C function? Do the R26-CreERT2;Vegf-Cflox/flox mice develop edema at E17.5-E18.5 after tamoxifen-administration at

E12.5 and E13.5?

Fig.1I-L and Suppl. Fig.1 refer to postnatal deletions between P1 and P4 and analysis of mice at P6. Fig.1K,L and Suppl. Fig.1 E,F both illustrate lack of effect on lymphatic vessels in the intestinal wall and should both be mentioned in the text p.6.

Fig.1M-U and Suppl. Fig.2 show very nice comparison between Vegf-C deleted and Vegf-C and D double-mutant tissues. Is Vegf-D upregulated in Vegf-C mutant intestine? If so what is the source of Vegf-D in intestinal tissue?
Is Vegfr3 deletion as severe in blocking intestinal lymphatic growth as deletion of both ligands? Suppl. Fig.3 A,B show effects after 3months deletion on lacteal growth, please also show lymph vessels in intestinal wall to allow at least qualitative comparison between loss of ligands and receptor. Also, does Vegfr3 deletion affect only lacteals or are other adult lymphatic beds affected as well?
Can the authors speculate why lacteals are particularly sensitive to Vegf-C-Vegfr3 inhibition? Do these vessels have a higher turnover rate compared to other lymphatics?

What is the evidence to show that regression of lacteals occurs by cell death (text p.9)?

Fig.2E SMA staining looks intriguing, why do the SMA+ cells extend like fibers into the villi? Wouldn't they be expected to surround blood vessels?

Suppl. Fig.4A: Please provide reference for WR1339 effect on lipoprotein lipase (text p10). What are effects on serum triglyceride levels in Vegf-C mutant mice if no WR1339 is used? One would expect that absence of lacteals should prevent lipid uptake even when lipoprotein lipase activity is normal. Is lipid uptake into enterocytes normal in Vegf-C mutant mice?

Fig. 3A: Is the weight of the Vegf-C mutant mice similar to that of control littermates on normal chow, i.e at the onset of high fat diet feeding? The Vegf-C mutant mice do gain weight on high-fat diet, although less than control littermates. This is surprising and the authors should discuss this finding.

Suppl. Fig.4B: on a mixed background, Vegf-C mutant mice on high fat diet gain as much weight as their littermate controls. Do these mice develop lacteals? If they don't develop lacteals, how does lipid get taken up? If development of lacteals is dependent on genetic background, this needs to be clarified.

Finally, one is somewhat surprised that mice appear healthy, despite lack of intestinal lymphatic vessels. The authors should discuss potential effects of lack of lacteals on general mouse health.

1st Revision - authors' response 22 September 2015

We would like to thank the reviewers for their valuable time spent to evaluate our manuscript.

Referee #1 (Remarks):

General

The manuscript by Nurmi H. et al. describes the phenotype of Vegfc gene deletion in adult mice. General deletion of Vegfc using the R26Cre-ERT2 driver mouse line surprisingly only affected intestinal lymphatic vessels, resulting in vessel atrophy, resulting in defective lipid absorption and increased excretion of dietary cholesterol and fatty acids. Furthermore, this perturbed intestinal lipid transport protected Vegfc-deficient mice from obesity when placed on a high-fat diet. The work presented here is of great interest for the EMBO Mol Med readership, and furthers our understanding of the factors that regulate lymphatic vessel maintenance and function in postnatal health.

Comments:

1) The statement on page 5 that Vegfc gene-deleted embryos "die between embryonic days (E) 15.5-17.5, soon after the first lymphatic endothelial cells emerge from embryonic veins" should be clarified in light of the fact that the first lymphatic precursors emerge from the cardinal vein at E10.5.

We agree with the Reviewer and have corrected this as follows: "In *Vegfc* gene-deleted embryos, lymphatic vessels sprouting from the major embryonic veins at embryonic day (E) 10.5 is arrested and the embryos die between E15.5 and E17.5 (5)." (page 5, paragraph 1).

2) What is the penetrance and severity of VCiΔR26 phenotypes for the various 4-OH tamoxifen administration regimens?

All VCiΔR26 mice have a very similar and consistent phenotype with 100% penetrance. The lymphatic vessel growth stops after 4-HO tamoxifen administration and edema is clearly visible.

3) Are any other lymphatic vascular beds affected during embryonic development as a result of deletion of Vegfc? Given the recent papers on lymphatics in the brain and heart, does loss of Vegfc affect lymphatics in these organs, and is development of these organs also affected?

The embryonic phenotype induced by deletion of VEGF-C is very robust and impairs lymphatic vessel growth and the overall development of the embryo. Analysis of organ development is confounded by the severe edema formation, making organ-specific conclusions impossible. In collaboration with Dr. Red-Horse we have recently published that VEGF-C is involved in coronary vessel formation, which precedes the effect on lymphatic vessels development in the heart (Chen et al., JCI 2014, PMID: 25271623). In our recent publication by Aspelund et al. we showed that *K14-VEGFR3-Ig* transgenic mice lack meningeal lymphatic vessel but do not have obvious defects in brain development (Aspelund et al. JEM 2015, PMID: 26077718).

4) For the LYVE1 staining of the lacteal (lymphatic) vessels in Fig. 1K,L, a second lymphatic marker would be beneficial to demonstrate that lymphatic vessels do not simply have reduced LYVE1 immunoreactivity as a result of loss of Vegfc.

We have now addressed this question with additional stainings in combining the well-known lymphatic vessel markers Prox1 and VEGFR-3 to show that the VCiΔR26 pups do not have lacteal vessels. However, strong VEGFR-3 staining is found in the developing blood vessels. We have now included these data in Supplementary Fig. 1.

5) On page 6, it is unclear what is meant by "six-day postnatal Vegfc deletion" where "no changes were seen in the lymphatic vessels that develop during the intrauterine period" (referring to S. Fig. 1E-H). In the micrographs provided, panel F appears to have a reduced lymphatic area, with thinner vessels that have less visible branch points (compared to S. Fig. 1E). Can this be clarified.

We have now re-analyzed the P6 lymphatic vessel density in the intestinal wall from new samples. The result confirms a significant reduction of the lymphatic vessels after *Vegfc* deletion (page 6, paragraph 1).

6) On page 7, it is stated that there were "thinner and shorter lacteal vessels with a reduced number of lymphatic endothelial cells", and "the slow lacteal regression after genetic deletion of Vegfc occurs primarily by the loss of LECs via cell death". How was this assessed?

No proliferation of the lacteal lymphatic endothelial cells was observed in adult WT mice in whole-mount staining for Lyve1 and EdU after six days of EdU labeling. The analysis included 3D imaging of over 20 lacteal vessels. A strong EdU signal was present in numerous other cells of the villi in the same samples. Thus a difference in the proliferation rate does not seem to explain the slow atrophy of the lacteal vessels.

Although the VEGFR2- and VEGFR3-blocking tyrosine kinase inhibitor induced blood vessel regression in the villi, as published (Kamba. et al., Am J Physiol PMID: 16172168), it did not induce regression of the lacteal vessels during two weeks of daily administration. Furthermore, we did not observe sprouting of lacteal vessels in adult villi. Although the very slow atrophy of lymphatic vessels makes it difficult to clarify the mechanism, for example, by staining of apoptotic cells, there is a clear decrease of lymphatic endothelial cells per villus in the Vegfc deleted mice (Page 7 and Suppl. Fig. 2A). We thus conclude by default that a slow cell death is leading to vessel atrophy in the Vegfc deleted mice.

7) The statement that "VEGF-D can partially compensate for the absence of VEGF-C" is intriguing. Is there an upregulation of VEGF-D in VCiΔR26 mice?

No *Vegfd* upregulation was observed after *Vegfc* deletion in the intestine. However, lack of VEGF-C should allow more effective binding of VEGF-D to VEGFR-3, thus revealing the effect of VEGF-D deletion in the absence of VEGF-C. We have added this argument and data in the manuscript (Page 7, end of the page).

8) Is the long-term use of anti-VEGFR3-targeted therapies likely to affect dietary uptake and the nature of intestinal lymphatic vessels? What are the likely consequences of prolonged targeting of VEGF-C (or VEGFR3) to limit intestinal lipid absorption? It would be interesting for the EMBO Mol Med readership to place these interesting findings in perspective.

We would like to thank the reviewer for this comment. We have now discussed the clinical relevance of our findings more thoroughly (End of Results and discussion)

Minor comments

9) Immunostaining for Prox1 (white) in Fig. 1A-D is rather difficult to see in the micrographs provided, and should be highlighted with visual cues (i.e. asterisks) or changed to another color.

In this figure we used Prox1 staining to highlight the lack of lymphatic valves as a demonstration of the impaired maturation of the collecting lymphatic vessels. We have added asterisks to Fig. 1A-D to better highlight the typical increase of Prox1 expression in the valves. Individual nuclei are too small to be clearly visible at this magnification irrespectively of the color we use.

10. Additional labels should be provided in Fig. 1 to clarify which panels are WT and VCiΔR26. Are panels C,E,F WT and D,G,H VCiΔR26?

We have modified the labeling of Figure 1 to improve clarity.

11. The quality of the brightfield micrographs in Fig. 2 can be improved. The SMA-positive SMCs are difficult to detect, with respect to the architecture of the vessel wall and surrounding tissues. Immunofluorescence labeling of the lymphatic endothelium, along with staining for SMA and Beta-

Gal may improve visualization of VEGF-C localization.

We have encountered technical difficulties in immunostaining of β-Gal, which limited us to colorimetric β-Gal staining. Even with this method, the signal in the villus is very weak. We have now improved the image quality and the visual cues of high magnification images.

12. The quality of the PECAM1 staining in Fig. 2E is poor, and it is difficult to discern the organization of blood vessels, especially compared to the micrographs in Fig. 3F.

We have now repeated the stainings and improved the quality of Fig. 2E.

13. The graphs in Fig. 3 lack a line delineating the X-axis, and it would be easier for the reader to follow if the black and red bars were labeled in each graph.

A second figure legend has been added above the panel G to improve the clarity.

14. On page 14, "CONFLICT OF INTREST" should be "CONFLICT OF INTEREST". For S. Fig. 2B, Title: "Inquinal" should be "Inguinal"

The typos have been corrected.

15. Scale bars are missing from Fig. 2B (plus inset), 2C (inset), 2D and 2E; S. Fig. 2E (skin).

Scale bars have been added to panels.

16. Some of the journal abbreviations in the references are not correctly formatted (#10, 22, 23).

We used the same formatting for all the references but because these papers have only one or two authors, they appear different.

Referee #2 (Remarks):

Nurmi et al expand analysis of a recently described conditional Vegf-C allele that allows inducible deletion of this critical lymphangiogenic growth factor. Generation and characterization of a conditional Vegf-C allele is an important addition to the toolkit available to study the function of this important growth factor and the findings should be of wide interest to the angiogenesis community.

The generation of the allele, and description of lymphatic aplasia in the embryonic skin was published in JCI 2014, here the authors focus on adult deletion of Vegf-C using a tamoxifen-inducible ubiquitous Cre driver line. They show that adult deletion of Vegf-C preferentially targets lacteals, which are terminal lymphatic vessels within the intestinal villi that are responsible for uptake of dietary fat. Interestingly, they show that absence of lacteals prevents dietary fat uptake and renders mice resistant to diet-induced obesity, without apparent deleterious effects on mouse health. Very little is known about the mechanisms controlling chylomicron uptake into lacteals, this paper shows for the first time that lacteals are indispensable for dietary fat uptake and that preventing lacteal growth may be a novel avenue to combat obesity.

I find the paper of great interest for Embo Molecular Medicine and have only a few minor comments that the authors should address before publication.

1) Fig.1: is inducible deletion of Vegf-C equivalent to global loss of Vegf-C function? Do the R26-CreERT2;Vegf-Cflox/flox mice develop edema at E17.5-E18.5 after tamoxifen-administration at

E12.5 and E13.5?

Indeed, inducible VEGF-C deletion will cause edema due to lack of lymphatic vessel, as we showed for the dermal lymphatic vessel in our recent publication (Aspelund et al JCI 2014, PMID: 25061878). In the current study, we focused primarily on *Vegfc* deletion in adults, although the conditional mouse model allows more detailed analysis of lymphatic vessel development in future studies. This will be interesting, given the recent discoveries on the mechanisms of lymphatic vessel development in various tissues (Martinez-Corral I et al., Circ. Res. 2015 PMID: 25737499, Stanczuk et al., Cell Reports 2015 PMID: 25772358, Klotz et al., Nature 2015 PMID: 25992544).

2) Fig.1I-L and Suppl. Fig.1 refer to postnatal deletions between P1 and P4 and analysis of mice at P6. Fig.1K,L and Suppl. Fig.1 E,F both illustrate lack of effect on lymphatic vessels in the intestinal wall and should both be mentioned in the text p.6.

We have now re-analyzed the P6 lymphatic vessel density in the intestinal wall from new samples. This allowed us to confirm that there is a significant reduction of the lymphatic vessels after *Vegfc* deletion (page 6, paragraph 1).

3) Fig.1M-U and Suppl. Fig.2 show very nice comparison between Vegf-C deleted and Vegf-C and D double-mutant tissues. Is Vegf-D upregulated in Vegf-C mutant intestine? If so what is the source of Vegf-D in intestinal tissue?

No *Vegfd* upregulation was observed after *Vegfc* deletion in the intestine. However, lack of VEGF-C should allow more effective binding of VEGF-D to VEGFR-3, thus revealing the effect of VEGF-D deletion in the absence of VEGF-C. We have added this argument and data in the manuscript (Page 7, end of the page). Unfortunately we are not able to localize *Vegfd* expression reliably in the intestine because the β-Gal construct inserted into the Vegfd KO mice is not expressed, for unknown reasons.

4) Is Vegfr3 deletion as severe in blocking intestinal lymphatic growth as deletion of both ligands? Suppl. Fig.3 A,B show effects after 3 months deletion on lacteal growth, please also show lymph vessels in intestinal wall to allow at least qualitative comparison between loss of ligands and receptor. Also, does Vegfr3 deletion affect only lacteals or are other adult lymphatic beds affected as well?

The phenotype in *Vegfr3* deleted mice is not as prominent as the phenotype of *Vegfc* or *Vegfc/Vegfd* deleted mice, likely due to the compensatory effect of VEGF-C binding to VEGFR-2. Interestingly, *Vegfr3* deletion induces regression of lymphatic vessels in the skin and trachea (presented in Zarkada et al. PNAS 2015 PMID: 25561555, Fig S7), yet it only affects the lacteal vessels in the intestine. Lymphatic vessel staining in the intestinal wall is now included in Supplementary Figure 4 and discussed in the manuscript.

5) Can the authors speculate why the lacteals are particularly sensitive to Vegf-C-Vegfr3 inhibition? Do these vessels have a higher turnover rate compared to other lymphatics?

Please see Reviewer 1, point 6.

Interestingly, we analyzed the intestinal lymphatic vessels from germ free mice and observed that the villus and lacteal vessels are elongated when compared with convectional mice (Figure below). This indicate that lacteal vessel have the capacity to remodel and to meet the need for proper nutrient absorption which is deficient in germ free mice. This property of lacteal vessels could explain why these vessels have special requirements VEGF-C/VEGFR-3 for maintenance.

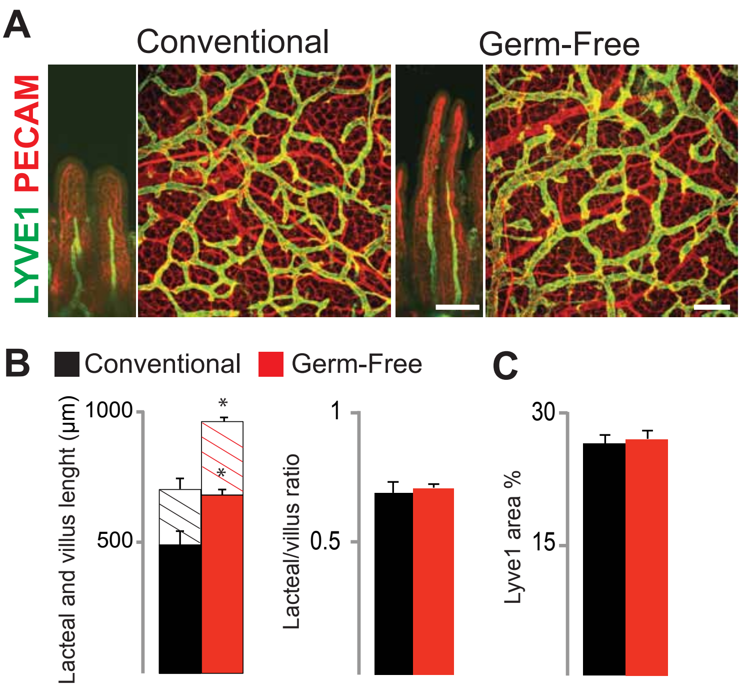

Figure legend. No differences in intestinal lymphatic vessels between conventionally housed and germ-free mice. (A) Representative images from villi and intestinal wall stained with LYVE1 (Green) for lymphatic vessels and PECAM1 (Red) for blood vessels (B) Quantifications form lacteal and villus lenghts, lacteal /villus ratio and intestinal wall lymphatic vessel area (LYVE1). Scale bar 200µm. n=3. *P<0.05. We kindly thank Professor Fredrik Bäckhed and Dr. Thomas Greiner from the University of Gothenburg for providing us samples for this analysis.

6) What is the evidence to show that regression of lacteals occurs by cell death (text p.9)?

Although the very slow atrophy of lymphatic vessels makes it difficult to clarify the mechanism, for example, by staining for apoptotic cells, there is a clear decrease of lymphatic endothelial cells per villus in the *Vegfc* deleted mice (Page 7 and Suppl. Fig. 2A). We thus conclude by default that a slow cell death is leading to vessel atrophy in the *Vegfc* deleted mice.

7) Fig.2E SMA staining looks intriguing, why do the SMA+ cells extend like fibers into the villi? Wouldn't they be expected to surround blood vessels?

It was shown before that these SMC fibers extend into the villus and are likely required for villus contraction during nutrient absorption (Hosoyamada and Sakai Anat. Embryol. 2005 PMID:16044319). The blood vessels in the villus are capillaries; they are not covered by SMCs.

8) Suppl. Fig.4A: Please provide reference for WR1339 effect on lipoprotein lipase (text p10). What are effects on serum triglyceride levels in Vegf-C mutant mice if no WR1339 is used? One would expect that absence of lacteals should prevent lipid uptake even when lipoprotein lipase activity is normal. Is lipid uptake into enterocytes normal in Vegf-C mutant mice?

A reference to WR1339 has been now added to the manuscript (page 10). The reviewer is right, the absence of lacteals should prevent lipid uptake even when lipoprotein lipase activity is normal, but the detection of uptake is confounded by the clearance of lipids. To determine more accurately the effects on lipid absorption, the clearance of lipids from plasma needs to be blocked.

© EMBO

Our preliminary data suggests that the lipid uptake to enterocytes is also reduced secondary to the shortening of the lymphatics. However, this requires a thorough study with isolated the epithelial cells.

9) Fig. 3A: Is the weight of the Vegf-C mutant mice similar to that of control littermates on normal chow, i.e at the onset of high fat diet feeding? The Vegf-C mutant mice do gain weight on high-fat diet, although less than control littermates. This is surprising and the authors should discuss this finding.

The weight of *Vegfc* deleted mice is similar to control littermates when fed a regular (low fat) diet. The lipid absorption in *Vegfc* deleted mice is significantly reduced but not completely blocked (Supplementary Figure 4A), explaining why the mice still gain weight when fed a high fat diet. We included this discussion in the manuscript (Page: 11, end of paragraph).

10) Suppl. Fig.4B: on a mixed background, Vegf-C mutant mice on high fat diet gain as much weight as their littermate controls. Do these mice develop lacteals? If they don't develop lacteals, how does lipid get taken up? If development of lacteals is dependent on genetic background, this needs to be clarified.

Mice on C57Bl/6J background are an established model for diet induced obesity and they seem to be more sensitive to changes in lipid absorption. The effects of obesogenic diets in mixed genetic backgrounds are difficult to interpret. The experiment on the mixed genetic background was performed in a non-SPF animal facility that can also affect the gut microbiota of the mice and the response to high fat diet. Unfortunately, we did not maintain the mice on the mixed genetic background to perform more detailed analysis in our current SPF facility, where the experiments on C57Bl/6J background were performed. It is also important to remember that constitutive Vegfc heterozygous mice do not survive in the C57Bl/6J background (Karkkainen et al., Nat Imm. 2004 PMID: 14634646), highlighting the importance of genetic background for the severity of phenotypes induced by VEGF-C deficiency.

11) Finally, one is somewhat surprised that mice appear healthy, despite lack of intestinal lymphatic vessels. The authors should discuss potential effects of lack of lacteals on general mouse health.

It is important to note that the deletion of *Vegfc* in adults induces lymphatic vessels atrophy, not a complete lack of lymphatic vessels. Therefore, lipid absorption still occurs in the Vegfc deleted mice at a lower rate. This should allow essential lipids and lipophilic vitamins to be absorbed sufficiently to have minimal effects on mouse health.

Recent evidence showed that immune cell trafficking through the lymphatics is normal even in severe cases of lymphatic vessel hypoplasia (Platt et al. 2013, PMID: 23530147).

Referee #3 (Comments on Novelty/Model System):

In this manuscript the Alitalo group expand on their previous excellent research on the mechanisms regulating lymphangiogenesis. Having previously demonstrated that VEGFC signaling is required for the migration and survival of lymphatic endothelial cells forming the lymph sacs, they now make use of an inducible VEGF-C knockout mouse model to investigate the role of VEGF-C in lymphangiogenesis during later stages of development. The main finding is that VEGF-C is required for developmental and early postnatal lymphangiogenesis in the intestine and plays a role in the long term maintenance of the lymphatic vessels in the intestine but not other organs (skin, trachea, lymph nodes) of adult animals. Additional interesting observations were that the phenotype of vegfc deletion was aggravated when Vegfd was also deleted and that Vegfc deleted mice showed defective lipid absorption rendering them more resistant to obesity and glucose intolerance when subjected to a high fat diet.

Referee #3 (Remarks):

The manuscript provides novel and interesting insights into the dynamic function of VEGFC in the regulation of the intestinal lymphatic vasculature. The maintenance function described for VEGFC in the intestinal lymphatic vasculature and its impact on lipid absorption represent exciting new aspects and opportunities for future studies in this field. The manuscript is concise and generally well written and the results are adequately presented in the figures. Having said this, on occasion the comparison between panels showing different experimental settings (embryonic vs postnatal vs long term deletion) different genetic backgrounds (pure vs mixed) in various organs (intestine, skin, trachea) and different types of analysis is somewhat confusing and hard to follow, especially when several variables change even in one figure or the results are split between the main figure and supplement. Nevertheless, the quality of the research is of a very high standard and the figures were carefully compiled.

Major Issues:

I have no major concerns.

Minor Issues:

1) Fig. 1: panels M-T are difficult to interpret without reference to panel U. Perhaps the authors could label these panels more clearly?

We apologize, but we did not find a better way to label the panels without masking the images with text.

2) Fig. S1A/B: could the authors include a brief comment in the text on the observation that the lymphatic vessel density was reduced in the skin?

This has been now added to the main text (Page: 6, paragraph: 1).

3) Fig. 2A: The difference in expression patterns of VEGFC vs VEGFR2 can be recognized but its not terribly clear. While I appreciate that technical difficulties are partly responsible, it might also help to show the tip of the villus to more easily relate the staining pattern to the precise position within the villus.
In Fig. 2B/C Given the technical difficulties in staining VEGFC, have the authors considered other markers of smooth muscle cells except SMA? Also labelling of the different structures stained (arterial SM, circ. muscle layer etc.) would be helpful for more rapid interpretation. The villus outline is hard to recognize without nuclear red counterstain (for obvious reasons), so perhaps this could be outlined instead?

Indeed, we encountered technical difficulties because immunofluorescent staining of *Vegfc* or β-Gal was unreliable. VEGF-C expression levels are low in the villus as indicated by the weak β-Gal signal that we observed.

In an effort to overcome these technical limitations we have changed the figure to better show the area covered by the higher magnification images and the staining patterns of *Vegfc* and *Vegfr2*. We also updated the labeling of the figure to improve clarity.

5) Fig. S2A: Again, the outline (tip to base) of the villus is not visible, making interpretation of the LYVE1 signal difficult. The Prox1 staining pattern shows a lot of background, and overall little

difference between the two panels -the stippled box appears to be placed rather arbitrarily.

Unfortunately we cannot improve the image quality because Prox1 staining gives a high background in the intestine and there are very few lymphatic endothelial cells per lacteal.

6) Fig. S2B The type of staining should be added to the figure.

Indeed, thank you, we have now added it.

7) Fig. S3C: The lack of lacteal regression in response to sunitinib is a very interesting and surprising finding, especially in comparison with the dramatic effects shown for VEGFR3 deletion. Given that Sunitinib is rather nonspecific, the use of more specific RTKIs might help to pinpoint the molecular signals involved. Has/could this been/be explored?

We treated the mice with Sunitinib for two weeks only whereas the VEGFR-3 deletion was for three months before analysis. This likely explains the difference in lacteal regression. Prolonged treatment with Sunitinib should eventually induce the lacteal regression, however the simultaneous strong blood vessel atrophy could severely impair mouse health.

8) Fig. S4D: please label which samples are serum and stool, respectively.

Fig. S4E: this is based on a pure background of mice, while the other data is from mixed background - please indicate this in the figure as well as in the legend.

We have clarified the labeling of the figure, as suggested by the Reviewer.

9) How do the authors explain the different response in terms of weight gain upon high fat diet in mixed (Fig. S4B) vs pure background (Fig. 3A) animals given similar GTT results upon VEGFC deletion? Have the authors looked for any differences in lymphatic vessel atrophy in the pure vs mixed background mice?

See answer to Reviewer 2, point 10.

10) The observation that VEGFc deleted mice appear to be resistant to high fat diet is of potential clinical value. In particular because in these mice only the intestinal lymphatic vessels are affected without compromising the remaining lymphatic system as underlined by the lack of significant accumulation of chyle.

Thank you for the suggestion; we have now included a discussion to highlight the clinical relevance of our findings (Page 11, paragraph 2).

© EMBO
